# Direct infusion mass spectrometry metabolomics dataset: a benchmark for data processing and quality control

**DOI:** 10.1038/sdata.2014.12

**Published:** 2014-06-10

**Authors:** Jennifer A Kirwan, Ralf J M Weber, David I Broadhurst, Mark R Viant

**Affiliations:** 1 School of Biosciences, University of Birmingham, Edgbaston, Birmingham, B15 2TT, UK; 2 Department of Medicine, University of Alberta, Edmonton, AB, Canada T6G 2EI; 3 NERC Biomolecular Analysis Facility – Metabolomics Node (NBAF-B), University of Birmingham, Edgbaston, Birmingham, B15 2TT, UK

**Keywords:** Data publication and archiving, Metabolomics

## Abstract

Direct-infusion mass spectrometry (DIMS) metabolomics is an important approach for characterising molecular responses of organisms to disease, drugs and the environment. Increasingly large-scale metabolomics studies are being conducted, necessitating improvements in both bioanalytical and computational workflows to maintain data quality. This dataset represents a systematic evaluation of the reproducibility of a multi-batch DIMS metabolomics study of cardiac tissue extracts. It comprises of twenty biological samples (cow vs. sheep) that were analysed repeatedly, in 8 batches across 7 days, together with a concurrent set of quality control (QC) samples. Data are presented from each step of the workflow and are available in MetaboLights. The strength of the dataset is that intra- and inter-batch variation can be corrected using QC spectra and the quality of this correction assessed independently using the repeatedly-measured biological samples. Originally designed to test the efficacy of a batch-correction algorithm, it will enable others to evaluate novel data processing algorithms. Furthermore, this dataset serves as a benchmark for DIMS metabolomics, derived using best-practice workflows and rigorous quality assessment.

## Background & Summary

Mass spectrometry based metabolomics is increasingly being used as a biomarker discovery tool in epidemiology and stratified medicine, for example to identify subgroups of patients with distinct mechanisms of disease or responses to drugs^1–3^. Such investigations typically require large-scale study designs in order to appropriately power the statistical analyses. Therefore, the metabolomics measurements are necessarily protracted over time and often require a multi-batch experimental design. This substantially increases the adverse impacts of analytical (or technical) variation that arises from the mass spectrometric measurements. Improvements in data processing algorithms to correct for such variation, and more generally to produce highly reproducible and robust mass spectral metabolomics data, represent a highly active area of research in metabolomics.

**Figure 1:**
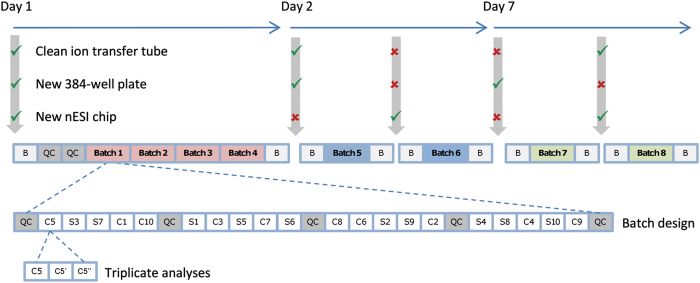
Experimental design of the eight-batch nESI direct infusion FT-ICR MS metabolomics study of polar extracts of cow (C, *n*=10) and sheep (S, *n*=10) heart muscle. All samples were analysed in triplicate in positive ion mode, with each analytical batch comprising of 20 biological samples and 5 equivalent QC samples; extraction blanks (B) were also analysed. The analytical batch was measured 8 times, across 7 days, while instrumental factors associated with normal mass spectrometer use were changed between batches to assess their impact on analytical variability. *Reprinted from* Kirwan, Broadhurst *et al.*^4^ *, Characterising and correcting batch variation in an automated direct infusion mass spectrometry (DIMS) metabolomics workflow, Analytical and Bioanalytical Chemistry 405(15): 5147–5157, with kind permission from Springer Science and Business Media.*

This dataset was collected in a study designed to measure the scientific validity and experimental reproducibility of a large, multi-batch direct infusion mass spectrometry (DIMS) metabolomics study of mammalian cardiac tissue extracts (Data Citation 1). The strength of the experimental design is the use of pooled quality control (QC) samples dispersed evenly across the multiple batches which enabled intra- and inter-batch variation to be corrected for using a software algorithm, Quality Control-Robust Spline Correction (QC-RSC), designed to map linear and non-linear temporal variation in the ‘biologically identical’ QC responses^4^. The effectiveness of this correction could then be assessed independently by examining the effects on repeated measurements of multiple biological samples. Specifically, the study comprises of three sample types: biological, QC and blanks. The 20 biological samples comprise of the polar metabolites solvent-extracted from 10 individual sheep’ and 10 individual cows’ cardiac tissues, together representing one analytical batch (Figure 1). Each batch of 20 biological samples, along with QC samples and blanks, was analysed repeatedly using nanoelectrospray direct-infusion Fourier transform-ion cyclotron resonance (FT-ICR) mass spectrometry, four times on day 1, twice on day 2 and twice on day 7, and hence every biological sample was measured in octuplet.

**Figure 2:**
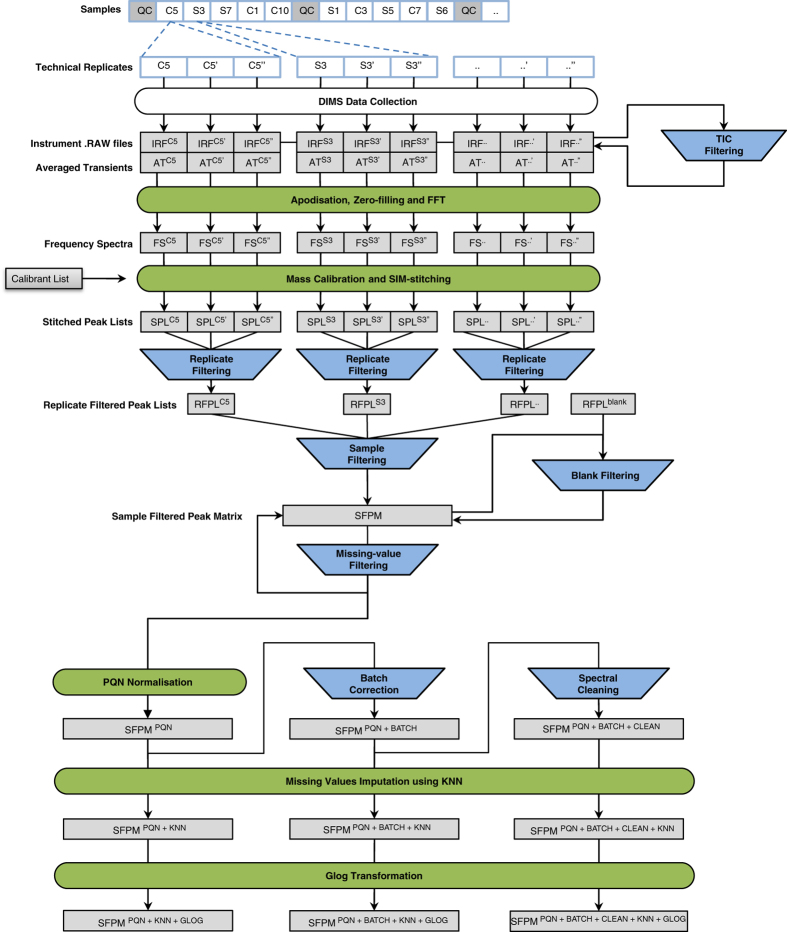
Data processing workflow for direct infusion FT-ICR mass spectrometry-based metabolomics dataset. All cow (C), sheep (S) and Quality Control (QC) samples were analysed in triplicate (e.g., for cow 5, analytical replicates C5, C5’ and C5’’) using direct-infusion mass spectrometry (DIMS). Samples with visual evidence of a poor electrospray current or with an outlying total ion count (TIC) profile were flagged as technical outliers. Data and metadata in the instrument .RAW files (IRF) and averaged transients (AT) were subject to apodisation, zero-filling and fast Fourier transformation (FFT). Next, the resulting frequency spectra (FS) were mass calibrated and SIM-stitched, which resulted in three stitched peak lists (e.g., SPL^C5^, SPL^C5’^ and SPL^C5’’^) for each sample. The peaks in each SPL were subject to four further filters in order to discard noise and retain only the more robust peaks, comprising of replicate filtering, blank filtering, sample filtering and missing-value filtering. These processing steps generated two datasets: a replicate filtered peak list (RFPL) for each sample, and a single sample filtered peak matrix (SFPM) for the whole study. The technical outliers as identified by the TIC-filtering were removed from the dataset immediately prior to sample filtering. The SFPM dataset was further processed using probabilistic quotient normalisation (PQN), QC-robust spline batch correction, and spectral cleaning. Finally, each of these three data matrices was subject to missing values imputation using KNN and then variance stabilisation using a generalised logarithm transformation. Light-gray boxes represent the different datasets, blue trapezoids represent the data filtering steps, and green ovals represent the remaining processing steps.

The analytical and computational methods used to collect and process this dataset serve as a benchmark for a DIMS metabolomics study, having been developed and optimised over the last eight years^4–14^ (Figure 2). Nanoelectrospray DIMS is increasingly being utilised in both metabolomics and lipidomics, benefitting from a rapid analysis time^15–17^, high technical reproducibility^15^, comparable prediction capabilities to liquid chromatography-mass spectrometry (LC-MS)^16^, and requires minimal sample biomass^15,18^. Known disadvantages, caused largely by the absence of chromatography, include ion suppression^17^ co-elution of metabolites into the mass spectrometer^19^ and the production of complex spectra that yield mass to charge ratio (*m/z)* and intensity values only, limiting metabolite identification to putative annotations at best^20^. Here the transient (time domain) data and metadata were collected and processed using the selected-ion-monitoring (SIM)-stitching method^8,10^, including Fourier transformation, internal mass calibration and spectral ‘stitching’ (Datasets 1–5). Next, several processing steps were undertaken to detect and then retain only high quality peaks that were present in the majority of samples, including the removal of peaks detected in the blank samples, and additional steps ensured that ill-behaved samples, for example with a high percentage of missing peaks, were removed (Datasets 6–7). The final stages of the workflow comprised of data normalisation, batch correction, missing value imputation and a generalised logarithm transformation (Datasets 8–10), preparing the mass spectral data for statistical analysis. We have included this breadth of data to maximise the re-use potential by the widest possible range of users, acknowledging that the raw and minimally processed data will be of greatest value to those experienced in mass spectrometry, comprising of Dataset 1: Instrument .RAW files, Dataset 2: Averaged transients (AT), Dataset 3: Frequency spectra (FS) and Dataset 5: Stitched peak lists (SPL). All datasets are stored in the MetaboLights open access data repository^21,22^.

While a number of methods exist for the quality assurance and quality control of metabolomics data^23^, their use is not yet widespread nor have these methods been developed into formal recommendations for the scientific community. Here we present some ‘best practice’ procedures for a DIMS metabolomics study to assess the final quality of the data produced, including quality assessment of analytical precision^6^ and multivariate quality assessment using QC samples^24,25^. Furthermore, our description of this dataset adopts the draft recommendations of the Metabolomics Standards Initiative^20^. In addition to its value as a benchmark DIMS metabolomics dataset, derived using ‘best practice’ workflows and validation, it has considerable re-use potential. This stems in part from the unusual experimental design, namely the high level of repeated measurements of each biological sample (*n*=8), the level of replication of biological samples per group (*n*=10), and the multiple analyses of one pooled QC solution throughout the study. While we have used this design to allow a robust examination of the benefits of a batch correction algorithm, others may wish to explore the development of additional or alternative signal processing algorithms by applying any filtering or correction to the QC samples and then independently measuring the effect on the reproducibility of the repeatedly-measured biological samples. Furthermore, by including all relevant datasets throughout the entire workflow, others can readily investigate novel signal processing algorithms at any stage of the data processing.

## Methods

### Experimental design

The datasets presented here arise from a direct infusion mass spectrometry (DIMS) based metabolomics study of mammalian cardiac tissue extracts. The study was originally designed to evaluate intra- and inter-batch variation in a DIMS metabolomics study. There are three types of sample: biological, quality control (QC) and blank. The 20 biological samples comprised of 10 individual sheep (S) and 10 individual cow (C) extracts, which together we classify as an analytical batch (Figure 1). QC samples were all derived from a single QC pool, produced as described in the Sample preparation section below, and were analysed at the beginning and end of each batch and after every five biological samples. The ten blank samples were all obtained from the same solvent that was used for reconstitution of the biological and QC samples. Blank samples were analysed at the start and end of most analytical batches. In addition, two QC samples were analysed at the start of the study to allow for equilibration^26^. Specifically, each batch of 20 biological samples was analysed repeatedly, four times on day 1, twice on day 2 and twice on day 7, and hence every biological sample was measured in octuplet in a non-random repeating order (Figure 1). Furthermore, every sample (biological, QC and blank) was analysed in triplicate (Figure 1) with each of these three analyses of a sample being termed replicate 1, 2 or 3. As indicated in Figure 1, no instrument variables were changed across the first four batches on day 1 to assess any potential instrument drift, and then, either one or two instrument variables were altered between subsequent batches on day 2 and day 7 (including changing the 384-sample plate, changing the electrospray chip, or cleaning the ion transfer tube in the inlet of the mass spectrometer). A unique aspect of this experimental design is that intra- and inter-batch variation can be corrected using QC spectra, as described below, and the quality of this correction assessed independently by examining the effects on repeated measurements of the 20 biological samples.

### Sample preparation

Methanol and water (both HPLC grade) were purchased from Scientific and Chemical Supplies Ltd. (Bilston, UK), and chloroform and formic acid (both HPLC grade) were purchased from Fisher Scientific (Loughborough, UK). Homogenisation tubes with ceramic beads were purchased from Stretton Scientific (Stretton, UK). Fresh sheep (*Ovis aries*; *n*=10) and cow (*Bos primigenius taurus*; *n*=10) heart tissue was obtained fresh from a local abattoir less than 4 miles away, packed on ice and driven back to the laboratory where it was frozen in liquid nitrogen. Portions of heart tissue were dissected and weighed (52 mg±2.6 mg) before storing at −80 °C until extraction. Since the purpose of the study was to assess the analytical reproducibility of a DIMS metabolomics study, no data was collected on the sex, breed or origin of the tissue supplied.

Low molecular weight metabolites were extracted from each cardiac tissue using a methanol/chloroform/water solvent system (final solvent ratio 2:2:1.8) as described previously^12^. Briefly, pre-weighed frozen tissue was homogenised in 8 ml/g of ice-cold methanol and 2.5 ml/g of ice-cold water in Precellys homogenisation tubes containing ceramic beads. The homogenate was transferred to 1.8 ml glass vials to which 8 ml/g chloroform and 4 ml/g water were added. Samples were vortexed and placed on ice for 10 min before the biphasic mixture was centrifuged at 1800-*g* for 10 min. After 5 min at room temperature, ten 15 μl aliquots of the polar layer were transferred into ten 1.8 ml glass vials, dried in a centrifugal evaporator (Thermo Savant, Holbrook, NY) and stored at −80 °C until analysis. Further tissue from the same anatomical region of the same twenty hearts was extracted as one pool and then aliquoted in 100 μl aliquots to make ten identical QC samples.

### FT-ICR mass spectrometry

All biological and QC samples were reconstituted in 80:20 methanol/water containing 0.25% (by volume) formic acid. Samples were centrifuged at 15000-*g* at 4 °C for 10 min to remove particulate matter. Metabolomic analyses were conducted in positive ion mode using a hybrid 7-T Fourier transform ion cyclotron resonance mass spectrometer (LTQ FT Ultra, Thermo Fisher Scientific, Bremen, Germany) with a chip-based direct infusion nanoelectrospray ion source (nESI; Triversa, Advion Biosciences, Ithaca, NY). Nanoelectrospray conditions comprised of a 200 nl/min flow rate, 0.3 psi backing pressure and +1.7 kV electrospray voltage controlled by ChipSoft software (version 8.1.0). Mass spectrometry conditions included an automatic gain control setting of 1×10^6^ and a mass resolution of 100,000 (at 400 *m/z*). Analysis time was 2.25 min (per technical replicate), controlled using Xcalibur software (version 2.0, Thermo Fisher Scientific). Spectra were collected using the SIM stitching method^10,14^, i.e., acquisition of seven overlapping selected ion monitoring (SIM) mass ranges between 70 and 590 Da in 100 Da windows, each with a 30 Da overlap with its neighbouring window. This SIM stitching method has previously been shown to increase metabolome coverage compared to a traditional wide-scan approach, while providing high mass accuracy and a rapid sample throughput. For each replicate of each sample, data was collected as both proprietary-software-processed .RAW mass spectral files (Dataset 1: Instrument .RAW files) and as multiple individual transient files (i.e., signal intensity in the time domain), discussed further below.

### Signal processing of mass spectra

Data processing steps have been reported as suggested by the Metabolomics Standards Initiative^27^. The principal processing steps are summarised in Figure 2. Initially, the instrument .RAW files were inspected manually to determine if any samples failed to electrospray, i.e., if the total ion count (TIC) dropped to zero. In the event of any single replicate suffering a sustained electrospray current failure, all three replicates for that sample were removed from the dataset. This equated to the removal of 9 samples spread randomly across the entire 8-batch study (but were included in MetaboLights as instrument .RAW files in Dataset 1). To further improve data quality, the TIC measurements were then examined in greater detail. For each of the three replicates per sample, this comprised of averaging ca. 15 intensities for each SIM window to create a representative seven-value TIC array of one median value per SIM window. The resulting dataset was analysed by principal components analysis (PCA) to identify technical outliers; 17 samples were deemed of poor quality based upon their outlying behaviour along the PC2 axis^4^. These were then flagged for removal from the dataset, a process called ‘TIC filtering’. Note that to maximise the re-use potential of this dataset, these 17 samples were maintained in Datasets 1–6 (since these outliers do not affect any other samples up to this point in the workflow), but removed from Dataset 7 onwards (to avoid them adversely affecting the amalgamation of sample spectra in the process of forming the whole-study data matrix).

Next, using custom written Matlab code (Data Citation 1); (The MathWorks R2009a, Natick, MA), the transient files containing the mass spectral data were averaged to produce Dataset 2: Averaged transients^8,10^. The metadata for each replicate, including the measured mass to charge (*m/z*) range, the TIC, the ion transfer time and the number of scans, is contained in the instrumental .RAW files from where it is collected and stored by the Matlab code, and used during subsequent stages of the processing. Then the averaged FT-ICR transient data were Hanning apodised, zero-filled once and Fourier transformed using the fast Fourier transform (FFT) algorithm to convert them from a time to frequency domain (Dataset 3: Frequency spectra). Peak-picking was then achieved using a signal-to-noise-ratio (SNR) threshold of 3.5:1, such that only peaks with a SNR value above this threshold were considered real, and retained in the dataset. Next, a calibration step was applied to convert the frequency domain (*f*) to *m/z* values using the relationship given by equation (1), where A and B are calibration parameters.

(1)m/z=Af+Bf2

A is dependent on the magnetic field in the ICR cell whereas B accounts for variations caused by space-charge effects. Internal mass calibration utilised a calibrant list of known-mass chemicals contained in the analysed samples (Dataset 4: Calibrant List). If no calibrants existed within a particular mass window, A and B parameters were taken from the values determined at the FT-ICR mass spectrometer’s weekly calibration, which are contained in the Instrument .RAW files (Dataset 1); this is a process called external mass calibration.

Next, for each individual replicate, the seven SIM windows were combined (or ‘stitched’) into a single file comprising frequency, intensity, resolution and SNR of each peak (Dataset 5: Stitched peak lists). ‘Replicate filtering’ was then applied where, for each sample, the *m/z* lists for the triplicate replicates were filtered to a single ‘combined’ peak list according to this criteria: a peak must be present in at least two out of three replicates (for that sample) to be retained (Dataset 6: Replicate filtered peak lists). This step primarily functions to remove random noise peaks from each individual sample^8^. ’Blank filtering’ was then applied to label and remove peaks from the biological samples that were also present in the blank samples; specifically, a threshold was set such that the peak was considered a contaminant if it had an intensity in the blank sample that was one-third or more of the intensity of the same peak in the biological samples. This allowed for high intensity peaks of biological origin to be retained, even if there was a much lower intensity coincident contaminant peak. Next, ‘sample filtering’ was applied, which amalgamates the individual biological sample spectra into a data matrix according to the following criteria: a peak must be present in at least 80% of the biological samples to be retained^8^.

**Figure 3:**
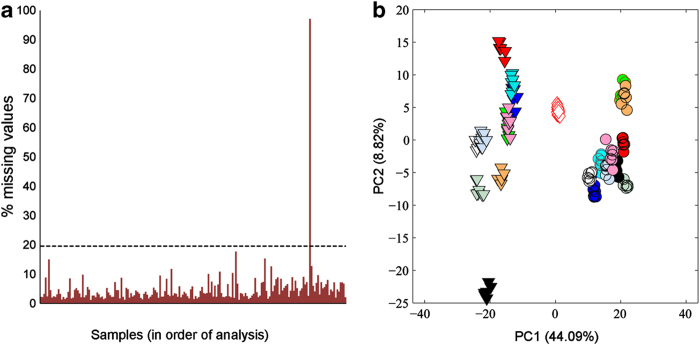
Assessing data quality in a multi-batch DIMS metabolomics study. (**a**) Bar chart showing the percentage of missing values in the mass spectrum of each QC and biological sample. The black dashed line represents the mean number of missing values plus two standard deviations, and is used as a 95% exclusion threshold. One sample (QC35) clearly exceeds the acceptability threshold for the number of missing values and therefore was excluded from the dataset. (**b**) PCA scores plot of the DIMS metabolomics dataset SFPM^PQN+BATCH+CLEAN+KNN+GLOG^ (PQN-normalised, batch-corrected, spectral cleaned, KNN missing value imputed and generalised logarithm transformed), colour-coded according to 20 biological samples and the QC samples. The tight clustering of the QC samples confirms that the analytical variation, both within and across all 8 batches, is small relative to the biological variation. Key: O sheep (multi-colours), ▽ cow (multi-colours), ◊ QC (red).

Together these processing steps yielded a Sample Filtered Peak Matrix (SFPM) of peak intensities with each row corresponding to a sample (QC or biological) and each column to a peak (*m/z*). While these settings ensure that every peak has non-zero intensities in at least 80% of the samples, there is no direct control over the percentage of missing values in any one sample. To improve data quality, samples with a high percentage of missing values were identified and removed (Figure 3a); a process called ‘missing-value filtering’, (which removed sample QC35), and then the ‘blank filtering’ and ‘sampling filtering’ processes were repeated to yield a more refined matrix (Dataset 7: SFPM). Subsequently the spectra were probabilistic quotient normalised (PQN)^28^, i.e., to reduce any variance arising from subtly differing dilutions of the biological extracts, which yielded Dataset 8a: SFPM^PQN^. Extensive statistical analyses were conducted on the SFPM^PQN^ dataset to assess the analytical reproducibility of the study, as described in the Technical Validation section below. Prior to multivariate statistical analysis (described in Kirwan, Broadhurst *et al.*^4^), the missing values in dataset SFPM^PQN^ were imputed using a K-nearest neighbour (KNN) method^5^ (Dataset 9a: SFPM^PQN+KNN^) and the matrix was prepared for multivariate statistical analysis by the application of a generalised logarithm (GLOG) transformation^7^ (Dataset 10a: SFPM^PQN+KNN+GLOG^) to minimise analytical variance across the peaks and reduce the likelihood that highly intense and variable peaks will dominate the PCA.

A ‘batch correction’ algorithm Quality Control-Robust Spline Correction (QC-RSC), which is based on an adaptive cubic smoothing spline algorithm^29^, was applied to the dataset to reduce intra- and inter-batch variation. QC-RSC is written in Matlab (version R2011a, The MathWorks, Natick, MA) and requires the Statistics and Curve fitting toolboxes. The batch correction software is available within MetaboLights (Data Citation 1). After the removal of any consecutive QC samples (e.g., QC01 and QC02) from the same batch to avoid overweighting the algorithm, QC-RSC was applied to the SFPM^PQN^ matrix (Dataset 8a), yielding Dataset 8b: SFPM^PQN+BATCH^, which was subject to missing value imputation (yielding Dataset 9b: SFPM^PQN+BATCH+KNN^) and then to the generalised logarithm transformation (yielding Dataset 10b: SFPM^PQN+BATCH+KNN+GLOG^).

Finally, and in conjunction with the batch correction algorithm, three ‘spectral cleaning’ algorithms were applied to the SFPM^PQN+BATCH^ dataset (yielding Dataset 8c: SFPM^PQN+BATCH+CLEAN^). First, a peak was removed if the relative difference between the median QC sample intensity compared to the median biological sample intensity was inconsistent between batches. This phenomenon is indicative of a failure to detect that peak in the QC samples, within one or more batches (tested using non-parametric Kruskal-Wallis test for comparison of batch medians, after signal correction, with a critical *P*-value of 0.0001). As such the peak as a whole must be considered unreliable and removed. In the second spectral cleaning algorithm, a peak was removed if the difference between the median QC sample intensity and the median biological sample intensity was relatively large (tested using non-parametric Wilcoxon Signed-Rank test, with critical *P*-value set empirically to 1×10^−14^). This phenomenon indicates that the QC samples are not in fact representative of the average of the biological samples, perhaps due to degradation. Finally, a peak was removed if its RSD^QC^ value was >20%; i.e. the analytical reproducibility of the peak was considered too high. This threshold is consistent with that used in the spectral cleaning of LC-MS based metabolomics datasets^26^. This final dataset was subject to the same processing and statistical analyses as previously described^4^ yielding Dataset 9c: SFPM^PQN+BATCH+CLEAN+KNN^ and Dataset 10c: SFPM^PQN+BATCH+CLEAN+KNN+GLOG^.

## Data Records

All data files, as detailed below, have been deposited to the MetaboLights metabolomics repository^21,22,30^ (accession number: MTBLS79, accessed *via* link: http://www.ebi.ac.uk/metabolights/MTBLS79). Associated metadata describing the samples, data collection, processing steps and the study as a whole have been captured using the ISA-creator package, available from the MetaboLights website (http://www.ebi.ac.uk/metabolights/download). ISA-creator, an open source metadata tracking tool, serialises the study contextual information in ISA-tab, a tab separated format^31^. The MTBLS79 archive contains the following ISA-tab files: (a) ‘i_Investigation.txt’—which holds project descriptors and summary information such as title, variables and description of the different data processing steps; (b) ‘s_MTBLS79.txt’—which describes the metadata related to the samples (e.g., organism, sample type, batch number); and (c) ‘a_MTBLS79_metabolite profiling_mass spectrometry.txt’—which captures the contextual data for the MS analysis (e.g., ion mode, type of mass spectrometry), from data acquisition through to the multiple processing steps (sample names, sample transformation names and derived data files), with each processing step matching the relevant datasets produced (see Figure 2 for more details). A link to the data repository is provided in the Data Citations section.

In total, 208 samples, each analysed in triplicate (totalling 624 .RAW data files) were uploaded using an ISA-tab format. These samples were used as input to three parallel data processing workflows of increasing complexity in the assay file. The rendering of this processing graph is presented in Figure 2. ISA-tab requires the unambiguous declarations of data input and data output for each of the many processing steps in the workflow. The result is a table that contains 624 records (3×208 samples) ensuring good traceability and the ability to review the processing steps, and allowing it to fully recapitulate the DIMS metabolomics workflow.

### Dataset 1: Instrument .RAW files (IRF)

This dataset is sited at MetaboLights (MTBLS79). It consists of 208 zip files containing the instrument .RAW files for the three replicates per sample that were collected for each blank, QC and biological sample that was analysed by DIMS metabolomics and processed using Xcalibur software (v.2.0.7). This dataset also includes the instrument .RAW files for the nine samples that were excluded from further processing due to poor electrospray current (e.g., batch 1: Sheep 3; batch 4: Sheep 4 and Sheep 9; batch 5: Cow 5, Cow 9 and Sheep 10; batch 6: Cow 1; batch 8: Cow 2 and Cow 6). Each file contains the total ion count, spectrum and metadata for each replicate. This dataset was used to provide metadata relating to each replicate including minimum and maximum *m/z* measured, total ion count, A and B calibration parameters for converting the frequency domain data into *m/z* values, specifications of the zero filling, ion injection time, filter index and the number of scans. Each zip file has been labelled as [batch number]_[animal][biological replicate number]__[dataset number]_[dataset name] (e.g. batch02_C04__dataset01_IRF.zip describes the instrument .RAW file for Cow 4 in batch 2). This method of labelling zip files is consistent throughout all other datasets described below.

### Dataset 2: Averaged transients (AT)

This dataset is sited at MetaboLights (MTBLS79). It consists of 199 zip files. Each zip file contains seven .mat files for each of the three replicates (i.e., 21 files in total) for each individual sample, and represents the output of the ‘Averaged transients’ command in our custom written SIMstitch code^10^. Each individual .mat file details the averaged transient data for one of the seven SIM windows collected for one of the replicate analyses. Contained within each .mat file are two files. SpecParam contains metadata including the A and B calibration parameters, TIC, and the starting and end *m/z* measured. The second file (called transient) contains the measured time domain values for that analysis. Each zip file has been labelled as [batch number]_[animal][biological replicate number]__[ dataset number]_[ dataset name]. Within each zip file, each .mat file has been labelled as [batch number]_[animal][Biological replicate number]_[run order]_[segment number (i.e. SIM window number)].

### Dataset 3: Frequency spectra (FS)

This dataset is sited at MetaboLights (MTBLS79). It consists of 199 zip files. Each zip file contains a single .mat file for each of the three replicates (i.e., 3 files in total) for an individual sample (e.g., batch01_C01_rep01_22_FS.mat). It represents the output of the ‘Processed transients’ command in our custom written SIMstitch code.

### Dataset 4: Calibrant list

This dataset is sited at MetaboLights (MTBLS79). It consists of a single.txt file detailing the calibrants used for the internal mass calibration of Dataset 3 to create Dataset 5: Stitched Peak Lists. The empirical formula plus adduct form is detailed in the left hand column with the theoretical *m/z* value listed in the right hand column.

### Dataset 5: Stitched peak lists (SPL)

This dataset is sited at MetaboLights (MTBLS79). It consists of 199 .txt files and 199 .mat files and represents the output of the ‘Stitch’ command in our custom written SIMstitch code^10^. Each text file (e.g. batch01_C01_rep01_22_SPL.txt) relates to an individual replicate of a sample after the seven SIM windows have been ‘stitched’ together to generate a single peak list per replicate. Each text file consists of four columns: ‘*m/z*’ the measured mass to charge ratio of the peak, ‘Intensity’ the intensity of the peak, ‘SNR’ the signal to noise ratio of the peak and ‘non-noise flag’ where 0 denotes a peak that has failed to pass the user defined SNR threshold and 1 denotes a true peak. The .mat files also represent individual replicates. Each .mat file (e.g., batch01_C01_rep01_22_SPL.mat) consists of two tree structures. The first specOut contains the data, and repeats many of the parameters already recorded in the text file. The second specOutParams is the same file as described in Dataset 2: Averaged Transients.

### Dataset 6: Replicate filtered peak lists (RFPL)

This dataset is sited at MetaboLights (MTBLS79). It consists of 199.txt files and represents the output of the ‘replicate filter’ command in SIMstitch. Each text file (e.g., batch01_C01__rep01_22__rep02_23__rep03_24_RFPL.txt) consists of the averaged composite of the three replicates of each sample, where a peak is only retained if it (a) satisfies a minimum SNR level and (b) is present in at least two out of three of the replicates. The file is arranged in three columns: ‘mz’ represents the measured mass to charge ratio of the peak, ‘intensity’ is the average intensity of the peak across the three replicates, and ‘num spectra (peak flagged)’ represents the number of individual replicate spectra the peak was detected in.

### Dataset 7: Sample filtered peak matrix (SFPM)

This dataset is sited at MetaboLights (MTBLS79) and represents the output of the processed transients, after extensive filtering of the data using our custom written SIMstitch code. It consists of an.xlsx file (i.e., Dataset07__SFPM.xlsx) with three tabs. The first tab ‘data’ consists of a data matrix of peak intensities with each row corresponding to a sample and each column corresponding to a peak. The second tab ‘meta’ consists of the metadata associated with the file including a sample index, sample type (QC or biological sample), batch, run order, sample_rep (where technical replicates of the same biological sample are assigned the same number, class (cow or sheep) and sample ID. The third tab ‘peak’ consists of the peak list that corresponds to the data matrix.

### Dataset 8a: SFPM^PQN^

This dataset is sited at MetaboLights (MTBLS79) and represents the PQN normalised version of Dataset 7 SFPM. It consists of an .xlsx file (i.e., Dataset08a__SFPM_PQN.xlsx) with three tabs. For more details on these tabs, see the description for Dataset 7 SFPM.

### Dataset 8b: SFPM^PQN+BATCH^

This dataset is sited at MetaboLights (MTBLS79) and represents the output of the batch corrected version of Dataset 8a SFPM^PQN^. It consists of an .xlsx file (i.e., Dataset08b__SFPM_PQN_BATCH.xlsx) with 3 tabs. The first tab ‘data’ consists of the batch corrected dataset of peak intensities with each row representing a sample and each peak a column. The second tab ‘meta’ has the metadata associated with the samples (see Dataset 7 SFPM for more details). The third tab ’peak‘ consists of a peak list, the median peak intensities (MPA) measured for each batch and the QC-RLSC parameter used for the correction of each batch.

### Dataset 8c: SFPM^PQN+BATCH+CLEAN^

This dataset is sited at MetaboLights (MTBLS79) and represents the output of the batch corrected and spectral cleaned version of Dataset 8a SFPM^PQN^. It consists of an .xlsx file (i.e., Dataset08c__SFPM_PQN_BATCH_CLEAN.xlsx) with 3 tabs. For more details on the contents of these tabs, see Dataset 8b SFPM^PQN+BATCH^.

### Dataset 9a: SFPM^PQN+KNN^

This dataset is sited at MetaboLights (MTBLS79) and represents the KNN missing value imputed version of Dataset 8a: SFPM^PQN^. It consists of an .xlsx file (i.e., Dataset9a__SFPM_PQN_KNN.xlsx) with three tabs. For more details on these tabs, see the description for Dataset 7: SFPM.

### Dataset 9b: SFPM^PQN+BATCH+KNN^

This dataset is sited at MetaboLights (MTBLS79) and represents the KNN missing value imputed version of Dataset 8b: SFPM^PQN+BATCH^. It consists of an .xlsx file (i.e., Dataset9b__SFPM_PQN_BATCH_KNN.xlsx) with three tabs. For more details on these tabs, see the description for Dataset 8b: SFPM^PQN+BATCH^.

### Dataset 9c: SFPM^PQN+BATCH+CLEAN+KNN^

This dataset is sited at MetaboLights (MTBLS79) and represents the KNN missing value imputed version of Dataset 8c: SFPM^PQN+BATCH+CLEAN^. It consists of an .xlsx file (i.e., Dataset9c__SFPM_PQN_BATCH_CLEAN_KNN.xlsx) with three tabs. For more details on these tabs, see the description for Dataset 8b: SFPM^PQN+BATCH^.

### Dataset 10a: SFPM^PQN+KNN+GLOG^

This dataset is sited at MetaboLights (MTBLS79) and represents the KNN missing value imputed and generalised logarithm (GLOG) transformed version of Dataset 8a: SFPM^PQN^. It consists of an .xlsx file (i.e., Dataset10a__SFPM_PQN_KNN_GLOG.xlsx) with three tabs. For more details on these tabs, see the description for Dataset 7: SFPM.

### Dataset 10b: SFPM^PQN+BATCH+KNN+GLOG^

This dataset is sited at MetaboLights (MTBLS79) and represents the KNN missing value imputed and GLOG transformed version of Dataset 8b: SFPM^PQN+BATCH^. It consists of an .xlsx file (i.e., Dataset10b_SFPM_PQN_BATCH_KNN_GLOG.xlsx) with three tabs. For more details on these tabs, see the description for Dataset 8b: SFPM^PQN+BATCH^.

### Dataset 10c: SFPM^PQN+BATCH+CLEAN+KNN+GLOG^

This dataset is sited at MetaboLights (MTBLS79) and represents the KNN missing value imputed and GLOG transformed version of Dataset 8c: SFPM^PQN+BATCH+CLEAN^. It consists of an.xlsx file (i.e., Dataset10c__SFPM_PQN_BATCH_CLEAN_KNN_GLOG.xlsx) with three tabs. For more details on these tabs, see the description for Dataset 8b: SFPM^PQN+BATCH^.

## Technical Validation

While a number of methods exist for the quality assurance and quality control of metabolomics data, their use is not yet widespread nor have these methods been developed into formal recommendations by relevant international organisations such as the Metabolomics Society. In 2007, the Chemical Analysis Working Group of the Metabolomics Standards Initiative took an important first step by recommending that data quality be assessed using standard error measures of the relative quantification of replicate analyses^20^, although these recommendations are clearly limited in scope. Here we present a series of procedures conducted on direct infusion mass spectrometry based metabolomics data to ensure high data quality, and further procedures to assess the final quality of the data produced. Some procedures have been developed over a period of several years in our respective laboratories and others have been derived from published literature and integrated into our laboratories’ workflows.

### Replicate measurements, QC and blank samples, and multi-step filtering

Given the chemical complexity of metabolomics samples, and the high sensitivity of modern analytical instrumentation, tens of thousands of 'peaks' are detected in .RAW direct infusion mass spectra of biological samples. Many of these, however, arise from chemical or instrumental noise, or are real yet irreproducible peaks that have been adversely affected by ion suppression during electrospray or ion–ion interactions within the mass spectrometer. Using modelling approaches, and based upon extensive empirical observations, we previously developed and characterised a series of filters to discriminate noise from real signals^8^. Furthermore, we previously conducted a thorough assessment of the occurrence of missing values within DIMS metabolomics datasets, as well as optimal imputation methods^32^. Most recently we have assessed the effects of measuring peak intensities in multi-analytical batch studies, and developed and implemented an algorithm to maximise the precision of repeated measurements of QC samples^4^.

The DIMS metabolomics study reported here was designed to allow all of these filtering steps to be included in the workflow. Specifically our experimental design includes the measurements of both QC and blank samples, as well as triplicate analyses of every sample (biological, QC and blank). The eight filtering steps that were applied in the processing pipeline (Figure 2), in the order indicated below, help to ensure that the data quality is high by meeting the following criteria:

1. the electrospray current did not ‘drop out’ and fail during data collection on the FT-ICR mass spectrometer;
2. none of the TIC profiles, representing the .RAW spectral data measured by the mass spectrometer, are outlying (see ‘TIC filtering’);
3. all peaks considered as detected in a sample are minimally measured in duplicate in that sample (see 'replicate filtering');
4. all peaks retained in the dataset occur in the majority of samples measured in the study (see 'sample filtering');
5. only peaks occurring at more than 3 times higher intensity in the biological samples relative to the blank samples are retained in the dataset (see ‘blank filtering’);
6. all samples have a relatively consistent number of peaks (see 'missing-value filtering');
7. any batch effects or temporal drifts in peak intensity, assessed on a peak-by-peak basis using the QC samples, are corrected for (see ‘batch correction’);
8. all peak intensities are measured reproducibly across batches within the QC samples, assessed on a peak-by-peak basis (see ‘spectral cleaning’).

Each of these filtering steps is described in more detail in the Methods section, including the thresholds applied during each process. The dataset presented here was measured with the purpose to develop the missing-value filter, batch correction and spectral cleaning. The beneficial effects of steps 7 and 8 are illustrated below, while the importance of including a missing-value filter to maintain the technical quality of the dataset is clearly illustrated in Figure 3a. Examining the number of missing values within each spectrum revealed that one QC sample had 2316 (97%) missing entries, far exceeding that of any other sample and above the quality control threshold, and therefore was removed.

### Quality assessment of analytical precision

Analytical reproducibility (or precision), within or between batches of DIMS data, can be assessed through the statistical analysis of QC samples. Specifically, the relative standard deviation (RSD) of the peak intensities for the QC sample population can be calculated for each peak. For biomarker studies, the FDA guidelines specify an RSD of <20% as an acceptable level of precision. Since metabolomics studies yield hundreds or thousands of peaks, this assessment of analytical reproducibility will yield hundreds to thousands of RSD values. Parsons *et al.*^6^ introduced a simple but pragmatic approach of reporting the median of these RSD values as a single summary statistic that characterises the analytical reproducibility in metabolomics. Hence the analytical reproducibility of the QC samples can be represented by a median RSD^QC^ value. A unique aspect of the experimental design presented here is that intra- and inter-batch variation was corrected using the QC spectra, and the quality of this correction was assessed independently by examining the effect on the eight repeated measurements of the biological samples; i.e., the analytical reproducibility of the biological samples have also been calculated across all 8 batches (here termed median RSD^biol^).

The median RSD^QC^ values are listed in Table 1, highlighting the reproducibility within each of the 8 analytical batches and across all 8 batches combined, at various stages of the data processing. For the PQN normalised (SFPM^PQN^) dataset, it is apparent that the spectra collected within each individual batch have a relatively high analytical reproducibility (7.4–11.5%). When the 8 batches of QC spectra are combined, however, the median RSD^QC^ increases substantially to 18.8% confirming that considerable batch-to-batch analytical variation occurs in this DIMS study. Following batch correction and spectral cleaning, the median RSD^QC^ decreases substantially to 8.6 and 8.2%, respectively, indicating the effectiveness of these processing steps for increasing the data quality. The median RSD^biol^ values are listed in Table 2 and also highlight the value of multiple data processing steps to maximise analytical reproducibility. We report an overall analytical reproducibility of 15.9%, measured as the median RSD^biol^ for the eight repeated measurements of the biological samples across 8 batches and 7 days of measurements. When compared against the FDA guidelines for biomarker studies, which specify an RSD of <20% as acceptable, we conclude that the optimised workflow presented here is fit-for-purpose for large-scale, high-throughput DIMS metabolomics studies.

### Final multivariate quality assessment using QC samples

As described in the Methods section, a single QC solution is prepared and then analysed repeatedly throughout the study. Clearly, any variation in the metabolic profiles of this QC solution arises solely from analytical variation, not from biological variation. The repeated QC measurements therefore represent a powerful and essential component of any metabolomics study. In addition to using these QC samples as an integral component of the batch correction algorithm (above) as well as to assess the analytical precision of every peak within the dataset (above), here we also used the QC samples for the multivariate quality assessment of the final dataset. This latter approach was first suggested by Sangster *et al.*^33^ and has subsequently been implemented into robust LC-MS based metabolomic workflows (e.g., Dunn *et al.*^26^). The multivariate approach most commonly used to assess this analytical variation involves conducting a PCA of the QC and biological samples, and then visualising the degree to which the QC samples cluster relative to the metabolic similarities and/or differences between the biological samples. Here, the multivariate comparison of the QC and biological metabolic profiles (for the optimally processed dataset SFPM^PQN+BATCH+CLEAN+KNN+GLOG^) clearly indicates that the analytical variation, both within and across all 8 batches, is small relative to the biological variation (Figure 3b).

## Usage Notes

As part of the complete dataset, all Matlab scripts mentioned here have been uploaded to MetaboLights, including a python script to provide full access to the *.mat files when Matlab is not available for the user. Basic guidelines have been included on the running order of the scripts and the authors can be contacted for further help if required.

Instrument .RAW files are accessible using Thermo specific software (i.e., Xcalibur software or MSFileReader) or other more generic toolkits such as ProteoWizard^34^.

It is noteworthy to re-iterate that the unusual experimental design presented here significantly increases the re-use potential for this DIMS metabolomics dataset, i.e., while we have used this design to allow a robust examination of the benefits of batch correction and spectral cleaning algorithms, others may wish to explore the development of additional or alternative signal processing algorithms by applying any filtering or correction to the QC samples and then independently measuring the effect on the reproducibility of the repeatedly measured biological samples. Furthermore, by including all relevant datasets generated throughout the processing of these direct infusion mass spectrometry measurements, others can investigate the effects of new signal processing algorithms at any stage of the workflow. For example, this dataset could be re-used to further investigate peak picking, de-noising of spectra or peak lists, normalisation algorithms, missing value imputation or batch correction methods, or indeed as a benchmark dataset to develop new statistical approaches.

## Additional information

**How to cite this article:** Kirwan, J. A. *et al.* Direct infusion mass spectrometry metabolomics dataset: a benchmark for data processing and quality control. *Sci. Data* 1:140012 doi: 10.1038/sdata.2014.12 (2014).

## Supplementary Material



## Figures and Tables

**Table 1:** Analytical precision of the DIMS metabolomics datasets presented as median RSD^QC^ values (%).

	**Dataset**		
	**SFPM** ^**PQN**^	**SFPM** ^**PQN+BATCH**^	**SFPM** ^**PQN+BATCH+CLEAN**^
Batch 1 (day 1)	8.7	7.3	6.9
Batch 2 (day 1)	9.7	8.3	7.8
Batch 3 (day 1)	7.4	6.3	6.0
Batch 4 (day 1)	10.0	8.3	7.8
Batch 5 (day 2)	8.5	4.9	4.6
Batch 6 (day 2)	11.5	8.3	8.0
Batch 7 (day 7)	9.0	7.2	7.1
Batch 8 (day 7)	8.5	7.0	7.1
All Batches (1–8)	18.8	8.6	8.2
Values have been calculated for each individual batch and across all eight batches, using the sample filtered peak matrices (SFPM) at various stages of the data processing workflow. The improvement in analytical precision following batch correction and spectral cleaning is clearly evident, as is the consistency of the technical variation between days 1, 2 and 7.			

**Table 2:** Analytical precision of the DIMS metabolomics datasets presented as median RSD^biol^ values (%).

**Sample**	**Dataset**		
	**SFPM** ^**PQN**^	**SFPM** ^**PQN+BATCH**^	**SFPM** ^**PQN+BATCH+CLEAN**^
Cow 1	17.3	17.0	15.0
Cow 2	17.8	17.7	15.6
Cow 3	18.2	17.9	15.7
Cow 4	16.7	18.3	16.4
Cow 5	18.3	16.5	15.4
Cow 6	14.0	16.4	14.8
Cow 7	20.4	20.0	17.7
Cow 8	21.9	20.8	17.7
Cow 9	16.9	16.3	14.2
Cow 10	20.2	19.0	16.9
Sheep 1	16.8	16.8	14.6
Sheep 2	18.5	16.9	15.4
Sheep 3	19.9	18.3	15.6
Sheep 4	18.0	17.4	14.9
Sheep 5	20.0	19.4	16.1
Sheep 6	19.9	18.9	17.1
Sheep 7	17.9	17.1	15.1
Sheep 8	20.2	21.0	18.7
Sheep 9	18.5	16.4	15.3
Sheep 10	18.2	17.1	15.1
Mean value	18.5	18.0	15.9
Values have been calculated for the eight repeated measurements of the biological samples, across all eight batches, using the sample filtered peak matrices (SFPM) at various stages of the data processing workflow.			
